# Wavelength Shifts in Hg^198^ As a Function of Temperature

**DOI:** 10.6028/jres.065A.049

**Published:** 1961-12-01

**Authors:** Sayeda H. Emara

## Abstract

The wavelength shifts for the green (5460A) and blue (4358A) lines emitted from an electrodeless discharge lamp of Hg^198^ have been studied as a function of the temperature of the water jacket of the source. The values of the wavelength shifts observed for the green and the blue lines are (8.5±3) 10^−6^ A/°C and (2±1) 10^−6^ A/°C, respectively.

## 1. Introduction

When satisfactory electrodeless discharge lamps containing a single isotope of mercury became available, several reports [1, 2, 3]^1^ were given recommending the use of this source as a secondary standard of wavelength.

The ease of construction and operation of electrodeless discharge lamps of Hg^198^ and the high intensity and relative sharpness of the widely spaced lines of this source have contributed to its wide general use for many interferometric and metrological purposes. There are no isotope shifts or hyperfine structure in the lines of Hg^198^ and because the atoms can be excited at relatively low temperature and vapor pressure, the wavelengths can be determined with great accuracy.

Two of the most commonly used spectral lines from this source are the green line (5460A) and the blue line (4358A). For visual adjustment of interferometers the green line of Hg^198^ is highly satisfactory and has been used extensively by several workers because it lies in the spectral region to which the normal eye is most sensitive.

Although an electrodeless Hg^198^ discharge lamp is convenient to use, the radiations emitted from this source suffer small wavelength shifts due to the pressure of the carrier gas and a change of vapor pressure of the mercury. Baird and Smith [4] have measured the shift due to the carrier gas pressure for a number of lines. They also pointed out the necessity for operating the source at a low temperature, preferably below 10 °C, [5] to reduce the asymmetric broadening and self absorption observed at higher temperatures. No quantitative measurement of this shift has as yet been reported.

Precision measurements of the wavelength of the green line have been reported by a number of laboratories. [6–11] These values are in disagreement. The discrepancies among the reported values might be due in part to the differences in the operating temperatures of the lamps. Consequently, a quantitative study of the effect of temperature was made. This paper reports the observed wavelength shifts of the green and blue lines as a function of the temperature of the water jacket of the source.

## 2. Experimental Details

The source was a Hg^198^ electrodeless discharge lamp of the type described by Meggers and Westfall [12]. It contained 1 mg of mercury. The carrier gas was argon at a pressure of 3 mm of mercury. The lamp was excited with a Raytheon microwave generator at a frequency of 2,450 Mc/s, and was operated in a jacket in which water was circulated. The radiation from this source illuminated a vacuum-enclosed Fabry-Perot interferometer with plate separation of 104.55 mm. The interference patterns were imaged on the slit of a quartz prism spectrograph by an achromatic lens of 75 cm focal length. The temperature of the water was measured to an accuracy of ±0.1 °C with a thermometer placed close to the outlet. When a constant water temperature was indicated, interferograms were taken with Kodak spectroscopic plates 103a–F. The exposure times were ten seconds and one minute at temperatures of 40 and 5 °C, respectively. Twenty-four exposures were made at alternate temperatures of the water jacket of 5 and 40 °C. This procedure was repeated for temperatures of 5 and 22.5 °C. The diameters of nine rings in the interference patterns for the green and blue lines were measured. From these measurements the fractional order at the center of each pattern was determined by using a procedure described by Meissner [13].

## 3. Results

The formula used for the calculation of the change in wavelength due to a change of temperature of the source is

$$\Delta \lambda =\frac{\lambda^{2}}{2t}\cdot \frac{\epsilon_{1}-\epsilon_{2}}{T_{2}-T_{1}}$$

where Δλ is the change in wavelength per degree centigrade; *T*_1_, *T*_2_ are the lower and higher temperatures of the water jacket of the source.

λ is the wavelength of the line being measured.

*t* is the plate separation of the interferometer.

*ϵ*_1_, *ϵ*_2_ are the average fractional orders measured at the specified temperatures.

The results obtained are summarized in the following table:

**Table:** 

Nominal wavelength	*T*_1_	*T*_2_	No. of determinations	Wavelength shift × 10^6^
				
*A*	*°C*	*°C*		*A*/*°C*
5460	5	40	10	+ 9.5 ± 3.0
5460	5	22.5	10	+ 7.5±2.5
			Average	+ 8.5±3
4358	5	40	10	+ 2.0±0.8
4358	5	22.5	10	+ 2.1±1.3
			Average	+ 2.0±1

The precisions indicated in the last column are average deviations.

The results indicate that the wavelength shift is nearly a linear function of temperature. Note, however, that the shift observed for the green line is about four times greater than that for the blue line and in the same direction. In both cases the wavelength increases with temperature. The green line also suffers to a greater extent from self absorption. These differences might be attributable to the fact that the lower state of the transition from which the green line arises is metastable. Therefore, for precision work using this source, the blue line at 4358A would appear to have advantages over the green line at 5460A.

## Footnotes

1 Figures in brackets indicate the literature references at the end of this paper.
